# PD-1 and its ligands are important immune checkpoints in cancer

**DOI:** 10.18632/oncotarget.13895

**Published:** 2016-12-10

**Authors:** Yinan Dong, Qian Sun, Xinwei Zhang

**Affiliations:** ^1^ Cell Immunology Lab, Tianjin Medical University Cancer Institute and Hospital, National Clinical Research Center for Cancer, Tianjin Key Laboratory of “Cancer Prevention and therapy”, Key Laboratory of Immunology and Cancer Biotherapy, Tianjin, China

**Keywords:** PD-1, PD-L1, PD-L2, T cell anergy, immune checkpoint blockade

## Abstract

Checkpoint programmed death-1 (PD-1)/programmed cell death ligands (PD-Ls) have been identified as negative immunoregulatory molecules that promote immune evasion of tumor cells. The interaction of PD-1 and PD-Ls inhibits the function of T cells and tumor-infiltrating lymphocytes (TIL) while increasing the function of immunosuppressive regulatory T cells (Tregs). This condition causes the tumor cells to evade immune response. Thus, the blockade of PD-1/PD-L1 enhances anti-tumor immunity by reducing the number and/or the suppressive activity of Tregs and by restoring the activity of effector T cells. Furthermore, some monoclonal antibodies blockading PD-1/PD-Ls axis have achieved good effect and received *Food and Drug Administration* approval. The role of PD-1/PD-Ls in tumors has been well studied, but little is known on the mechanism by which PD-1 blocks T-cell activation. In this study, we provide a brief overview on the discovery and regulatory mechanism of PD-1 and PD-L1 dysregulation in tumors, as well as the function and signaling pathway of PD-1 and its ligands; their roles in tumor evasion and clinical treatment were also studied.

## INTRODUCTION

Under normal physiological conditions, the major function of PD-1 is to inhibit effector T-cell activity and enhance the function and development of Tregs, which inhibit T-cell responses and prevent overstimulation of immune responses in peripheral tissues [1, 2]. The PD-1 pathway can protect the host against autoimmunity [3].

PD-1 pathway plays a key role in the regulation of antifungal and virus immunity [4]. PD-1 knockout (Pdcd1^−^/^−^) mice can lead to tissue sensitive to infection or late onset autoimmune disease with variable incidence depending on the background strain carrying the PD1 null animal [5-7]. Whereas, blockade of PD-1 enhances both proliferation of memory B cells and expansion of virus-specific CD8 T cells during chronic simian immunodeficiency virus (SIV) infection in macaques [8]. In persistently infected mice lacking CD4 T-cell help, blockade of the PD-1/PD-L1 inhibitory pathway had a beneficial effect on the ‘helpless’ CD8 T cells, restoring their ability to undergo proliferation, secrete cytokines, kill infected cells, and decrease the viral load [9].

Although tumor cells express numerous antigens, tumor evades T-cell responses and host immunity through negative regulators of the immune system, such as programmed death-1 (PD-1), programmed cell death-ligand-1 (PD-L1)/programmed cell death-ligand-2 (PD-L2), cytotoxic T lymphocyte antigen 4 (CTLA-4), T-cell immunoglobulin mucin 3 (TIM3), 2B4, the B and T lymphocyte attenuator (BTLA), and lymphocyte-activation gene 3 (LAG3) [10-12]. Among these regulators, CTLA-4 is a type 1 transmembrane glycoprotein mainly expressed on activated T cells. CTLA-4 inhibits T-cell function through intracellular signaling regulation *via* T-cell receptor (TCR) and CD28 in tumors [12, 13]. LAG3 (CD223) is a type I membrane glycoprotein of the immunogloblin (Ig) superfamily expressed in several different cell types, such as plasmacytoid dendritic cells (DCs), B cells, natural killer T cells, γ and δ T cells, exhausted CD8+ T cells, and regulatory T cells (Tregs). Association of LAG3 with PD-1 inhibits signaling passway in T-cell [12, 14]. TIM3 is a transmembrane molecule associated with CD8+ T-cell dysfunction and exhaustion. TIM3 is overexpressed on Tregs in tumor microenvironment. Tregs is related to ovarian tumor size. Blockade of TIM3 restores the inhibitory functions of tumor-infiltrating Tregs [15]. PD-1 and PD-L1/PD-L2 are identified as immune checkpoints that inhibit effector T-cell activity [1, 16].

PD-L1 is overrepresented in the presence of tumor and promotes immune evasion and growth of tumor by suppressing T-cell response [17]. PD-1/PD-L1 plays critical roles in cancer immunology, and blocking antibodies against this receptor provide benefits in clinical trials, with the first of this class recently approved by the *Food and Drug Administration* (FDA) to treat patients with refractory malignancies [16]. Recently, blockade of PD-1/PD-L1 has been found to treat effectively cancer by enhancing immunity. Several studies on Abs blockade of the PD-1 receptor (nivolumab, MK3475, or combination of nivolumab with the anti-CTLA4 checkpoint inhibitor ipilimumab) have improved survival profiles and acquired high response rates in several solid tumors [18-22]. In melanoma refractory to targeted therapy, pembrolizumab which is a humanized monoclonal IgG4-kappa isotype antibody against PD-1 induced overall response rates (ORRs) of 21%-34%. Among the patients with refractory non-small cell lung cancer (NSCLC), pembrolizumab induced ORRs of 19%-25%. On the basis of these results, pembrolizumab was approved by the USA FDA to treat advanced melanoma and NSCLC [23].

The function of PD-1 in peripheral tolerance and anti-tumor immune response is well established. Moreover, blockade of the PD-1 pathway has achieved good effect on restraining tumor. However, the exact mechanism of dysregulation of PD-1 and its ligands is still unknown. In addition, the manner of PD-1 ligation exerting its effects on specific signaling targets and how these altered signaling events affect T-cell function are yet to be completely understood.

## PD-1 AND THE REGULATION OF PD-1 EXPRESSION

PD-1 (also called CD279) was first isolated from 2B4.11 (a murine T-cell hybridoma) and interleukin-3 (IL-3)-deprived LyD9 (a murine hematopoietic progenitor cell line) by using subtractive hybridization technique [24]. PD-1 is encoded by the Pdcd1, which is located on chromosome 2 (*2q37*) [25-27]. PD-1 is one of the member of B7/CD28 family [28, 29]. PD-1 is also a 288 amino acid (55 kDa) type I transmembrane protein of the immune globulin superfamily, comprising an extracellular N-terminal IgV-like domain, a transmembrane domain, and a cytoplasmic tail [24, 26, 30]. There are two tyrosine residues in the cytoplasmic tail of PD-1; the N-terminal of which is involved in a sequence defined as the immunoreceptor tyrosine-based inhibitory motif (ITIM, I/L/VXYXXL/V); the C-terminal tyrosine is engaged in a sequence defined as immunoreceptor tyrosine-based switch motif (ITSM, TxYxxL) [24, 25, 31, 32]. The amino acid sequence around the C-terminal tyrosine (TEYATIVF) of PD-1 is well conserved between mouse and human and is related to SHP-1 and SHP-2. Whereas, the N-terminal tyrosine of PD-1 does not associate with either SHP-1 or SHP-2 [33].

PD-1 can promote the development, immunity evasion, and prognosis of several kinds of solid tumor, such as NSCLC, melanoma, breast cancer (BC), and renal cell carcinoma (RCC) [34]. Thompson's research showed that PD-1 was expressed in 56% of nephrectomy specimens of patients with RCC. PD-1 is also expressed in the T cells rather than in RCC tumor cells. Furthermore, the expression of PD-1 was associated with tumor stage, the presence of necrosis or sarcomatoid differentiation, and poor 5 year survival rate [35]. In classical Hodgkin lymphoma (cHL) and mediastinal large B-cell lymphoma, the extended PD-1 ligand/9p24.1 amplification region contains the Janus kinase 2 (JAK2) locus. JAK2 amplification promoted protein expression and activity, specifically inducing PD-1 ligand transcription and enhancing sensitivity to JAK2 inhibition. Therefore, PD-1 ligand/9p24.1 amplification is a disease-specific structural alteration that increases both the gene dosage of PD-1 ligands and their induction by JAK2, defining the PD-1 pathway and JAK2 as complementary rational therapeutic targets [36]. Programmed death 1 expression in the peritumoral microenvironment is an independent prognostic factor for Overall Survival (OS) of patient of cHL and is related to poor prognosis in cHL [37]. Similarly, epithelial-originated malignancy patients with PD-1 positive expression on TILs exhibited significantly shorter OS than the PD-1 negative group [38].

PD-1 is inducibly expressed on activated immune cell types including CD4+ T cells, CD8+ T cells, B cells, natural killer T cells, activated monocytes, DCs, macrophages [10, 28, 29, 39]. Moreover, PD-1 is selectively upregulated in T cells because of persistent exposure to antigens; thus, the expression of PD-1 in T cells is one of the makers of exhausted T cells [40-42]. A few mechanisms are involved in PD-1 expression regulation [43, 44]. Two upstream conserved regulatory regions of PD-1 gene termed as conserved regions B and C (CR-B and CR-C) exist. CR-B and CR-C are hypersensitive to DNase I and are important for PD-1 expression. CR-C was reported to contain a nuclear factor of activated T cells (NFAT) site, which is important for the transcriptional expression of Pdcd1, whereas, the role of CR-B is yet to be known [45]. In CD4+ and CD8+ T cells, the transcriptional activator nuclear factor of activated T cells c1 (NFATc1; also known as NFAT2) binding to CR-C and c-Fos binding to a site located in CR-B enhance the expression of PD-1 after TCR stimulation during the initial phases of Ag recognition [46, 47]. Xiao et al. identified that tumor-infiltrating T cells significantly upregulated the expression of the activator protein 1 (AP-1) subunit c-Fos. C-Fos (AP-1) directly binds to CR-B in the Pdcd1 (gene encoding PD-1) proximal promoter which increases PD-1 expression and enhances antitumor T cell function and restrained tumor growth [48, 49]. Bally et al. identified that NF-ΚB p65 binds to a region which located upstream of PD-1 gene in CR-C and enhances PD-1 expression following stimulation of macrophages with lipopolysaccharide (LPS) [50].

The 55 kDa src kinase-associated protein (SKAP55) and the adhesion and degranulation promoting adaptor protein (ADAP) are located at the killing synapses between CD8+ CTLs and tumor cells [51, 52]. Specifically, ADAP binds to SKAP55 and stabilizes its expression at protein level. Most importantly, ADAP-SKAP55 enhances PD-1 expression in a Fyn-, Ca2+-, and NFATc1 manner. ADAP-SKAP55 module enhances both total and activated NFATc1, which enhances PD-1 expression by binding to the promoter of PD-1. The ADAP-SKAP55-PD-1 pathway represents a “self-control” mechanism to control T-cell activation and adhesion precisely [46].

**Figure 1:**
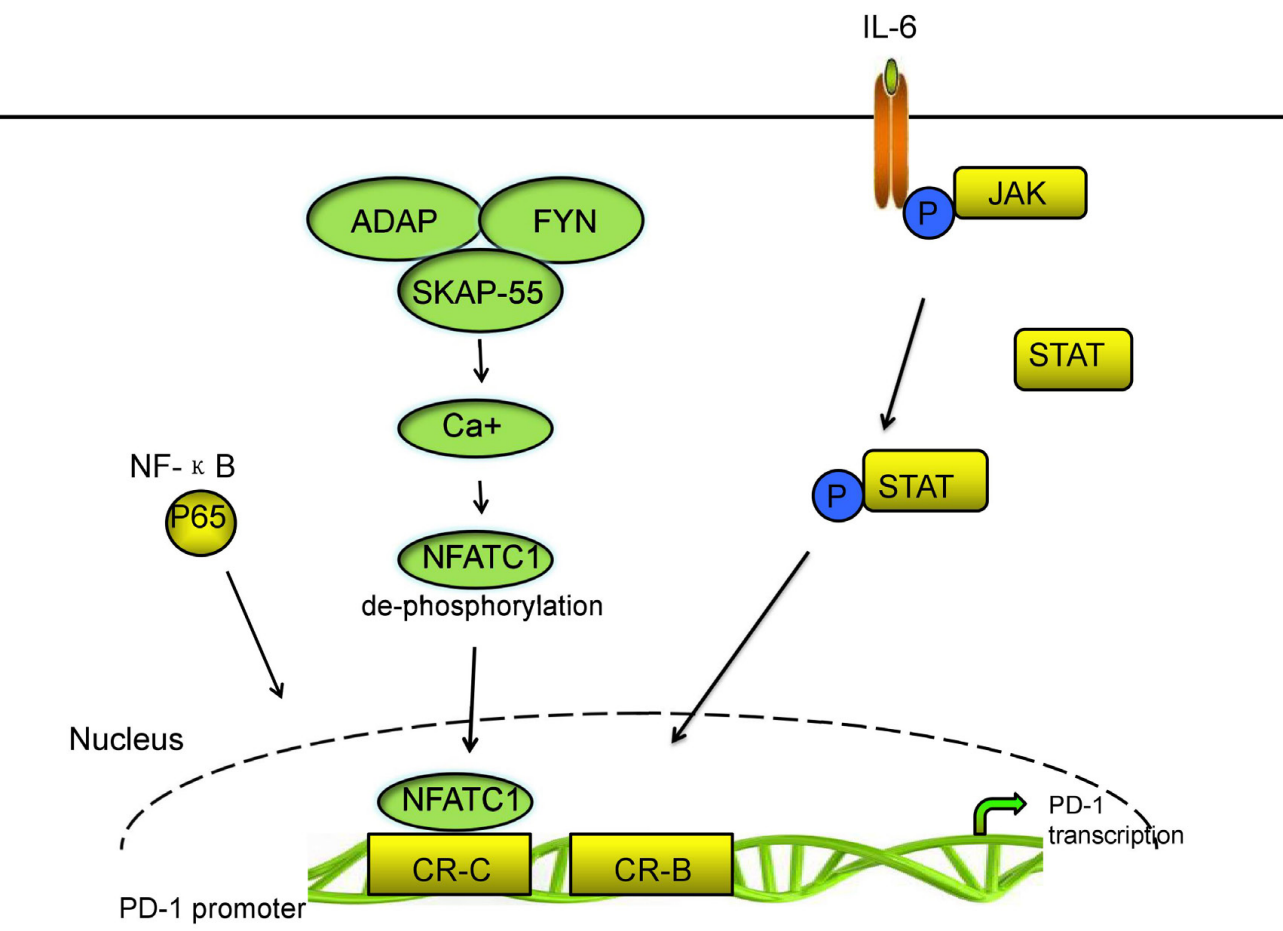
The main signal pathways of PD-1 transcriptional regulation.

Multiple cytokines, such as the common γ-chain family (IL-2, IL-7, IL-15, and IL-21) and type I IFNs (IFN- and IFN-β) can also upregulate PD-1 expression [53]. However, with the exception of IFN-α inducing responses from an interferon-stimulated regulatory element (ISRE) located in CR-C, no direct effect of cytokine-induced factors regulating Pdcd1 gene expression has been shown [49]. Kato et al. demonstrated that the concentration of IL-6 was in a high level, which is correlated to poor growth of cytomegalovirus (CMV)-specific T cells and high PD-1 expression on CMV specific T cells, and that disruption of IL-6 or the IL-6 receptor (IL-6R) interaction recovered CMV-specific T-cell growth. IL-6 and IL-12 induce the signal transducer and activator of transcription (STAT) activity STAT3 and STAT4, respectively, *via* the JAK family of proteins. STAT activity could change the chromatin structure of Pdcd1 and increase the PD-1 expression in splenic CD8 T cells. The NFATc1/STAT regulatory regions interact with the promoter region of the Pdcd1 gene and increase PD-1 expression following cytokine stimulation. Austin et al. found that Pdcd1 was regulated by distal elements, which is a non-biased approach employed across the murine Pdcd1 locus. Their group also found four novel distal regulatory regions. Two of these elements is located on the side of CCCTC-binding factor (CTCF). The third element, located upstream of CR-C, bound NFATc1 and STAT3 or STAT4 in response to TCR and IL-6 or IL-12 signaling, respectively. The final region, located close to the downstream CTCF site also bound NFATc1 and STAT3 or STAT4. Each of the novel NFAT/STAT elements interacts with the Pdcd1 promoter region and the chromatin structure of each regulatory region is altered in response to T-cell activation and cytokine stimulation in CD8 T cells, demonstrating that NFAT/STAT elements is associated with PD-1 expression [49, 54]. Vascular endothelial growth factor-A (VEGF-A) promotes PD-1 expression and other inhibitory checkpoints, which are involved in exhaustion of vascular endothelial growth factor receptor (VEGFR) expressing CD8+ T cells *in vitro*. Voron et al. identified that blockade of VEGF-A-VEGFR was sufficient to decrease PD-1 expression in intratumoral CD8+ T cells. Sunitinib, a multitarget tyrosine kinase inhibitor (TKI) that inhibits VEGFR1, R2, R3, platelet-derived growth factor receptors, and stem cell factor receptor, has been shown to suppress PD-1 expression at the mRNA level in tumor-infiltrating T cells [43, 55]. In addition, endogenous transforming growth factor-β (TGF-β) is also involved partially in PD-1 expression through TCR activation in T cells [56]. The main signaling pathways of PD-1 transcriptional regulation are shown in Figure 1.

By contrast, some transcriptional repressors inhibit PD-1 expression [47]. T-bet binds directly to Pdcd1 within a region of ~500 bp upstream of the transcriptional start site, near or within the CR-B region. This condition directly represses Pdcd1 transcription. When the antigen is persisted, T-bet expression is decreased in T cells; thus PD-1 expression is increased and functions of T cells are inhibited [45]. Meanwhile, the repressive effect of T-bet on PD-1 expression is not enhanced when T-bet is overexpressed. This condition indicates that other factors may also participate in inhibiting PD-1 expression. The B lymphocyte-induced maturation protein 1 (Blimp-1), which is encoded by the prdm1 gene, represses PD-1 gene expression through a feed-forward repressive circuit. On one hand, Blimp-1 binds to a site between CR-B and CR-C of the PD-1 gene to inhibit directly PD-1 gene transcription. On the other hand, Blimp-1 inhibits the expression of NFATc1 and displaces it from CR-C, leading to downregulation of PD-1 expression [47].

## PD-1 LIGANDS AND THE REGULATION OF THEIR EXPRESSION

PD-1 is involved in immune tolerance by suppressing activated immune cells via interaction with its ligands. Two known ligands of PD-1 are PD-L1 and PD-L2 [3, 29, 57]. B7-H1 was originally named as the first gene homolog of B7 molecules, and B7-H1 was renamed as PD-L1 after it has been identified as the first ligand of the receptor PD-1 (CD279) in the murine system [58]. PD-L1 gene is located in chromosome *9p24* [36, 59]. PD-L1 is a 290-amino-acid transmembrane glycoprotein [58, 60]. The second known counter-receptor of PD-1, called B7-DC or PD-L2, is also a member of the B7 family [58]. Hino et al. indicated that the degree of PD-L1 expression was correlated to the vertical growth of primary tumors in melanoma. Furthermore, multivariate analysis demonstrated that the survival rate of the PD-L1 high-expression patients was remarkably lower than that of the low-expression patients with stage II melanoma, which indicated that PD-L1 expression was an independent, poor prognostic factor for malignant melanoma [61]. PD-L2 may lead to local cytokine production that is beneficial to the tumor cells [62]. PD-L1 and PD-L2 play different roles in immune regulatory process although both are ligands of PD-1 [63]. PD-L1 inhibits T-cell function in peripheral tissues, whereas PD-L2 suppresses immune T-cell activation in lymphoid organs. PD-L2 inhibits type 2 T-helper (Th-2) lymphocytes, but its role is yet to be fully understood [53, 64].

PD-L1 is expressed on immune cells, including T cells, B cells, DCs, and macrophages [3, 57]. PD-L1 is overrepresented on several types of solid tumor cells, such as glioblastoma multiforme, NSCLC, and some hematologic malignancies [34]. Unlike PD-L1, which is expressed broadly, the expression of PD-L2/B7-DC is limited. PD-L2 is expressed mainly on antigen-presenting cells (APCs), including macrophages and myeloid DCs, and non-hematopoietic tissues, such as the lung [62, 63]. PD-L1 and PD-L2 are expressed on Respiratory tract epithelial cells (BEAS-2B cells). Moreover, the expression of respiratory tract epithelial cells is upregulated by respiratory tract virus infection or treatment with interferon-γ (IFN-γ) and IL-4. PD-L1 was moderately expressed, and PD-L2 was weakly expressed in unstimulated NCI-H292, BEAS-2B, and A549 cells [65]. Similarly, Kan-o et al. identified that polyinosinic-polycytidylic acid (poly IC) upregulates the expression of B7-H1 *via* activation of the NF-κB. Poly IC increases the generation of reactive oxygen species, which enhances the activation of PI3Kδ and NF-κB. In addition, poly IC-induced upregulation of B7-H1 was observably suppressed by a pan-PI3K inhibitor and partially by an inhibitor or a small interfering (si)RNA for PI3Kδ in BEAS-2B cells [66].

Both PD-L1 mRNA and protein can be upregulated by cytokines produced by infiltrating immune cells, such as IFN-γ, IL-4, IL-10, growth cell stem factors, bacterial LPS, and VEGF [10, 26, 44, 59, 67]. Several pathways exist that IFN-γ increases PD-L1 expression. Abiko et al. found that IFN-γ secreted by CD8+ lymphocytes upregulates PD-L1 in ovarian cancer cells and promotes progression of ovarian cancer. In mouse models, suppressing IFNGR1 (IFN-γ receptor 1) remarkably reduced the level of PD-L1 expression in tumor cells. By contrast, the injection of IFN-γ into subcutaneous tumors increased PD-L1 expression and promoted tumor growth [68]. Moreover, IFN-γ or toll-like receptor (TLR) stimulation upregulated PD-L1 expression in blast cells from patients with acute myeloid leukemia through MEK/ERK- and MyD88/TRAF6 pathway [69]. Chen et al. demonstrated that protein kinase D isoform 2 (PKD2), which is induced by IFN-γ, is an important regulator of PD-L1 expression in human oral squamous carcinoma cells. Inhibition of PKD2 activation not only suppresses PD-L1 expression and enhances an anti-tumor effect but also reduces drug resistance during chemotherapy [69]. By contrast, natural killer (NK) cell activation and secretion of IFN-γ significantly enhanced PD-L1 expression by activating JAK1, JAK2, and STAT1 in tumor cells. Inhibition of JAK pathway activation abrogates increased PD-L1 expression, which enhanced sensibility of tumor cells to NK cell activity [70]. Maine et al. identified that the concentrations of IL-10 and TGF-β in ascites were higher in patients with malignant ovarian tumors than with benign/border-line ovarian tumors. Both IL-10 and TGF-β can increase PD-L1 in monocytes *in vitro*. Blocking IL-10 with a neutralizing antibody reduced PD-L1 expression [71].

PD-L1 can be upregulated not only by some inflammatory cytokines but also by constitutive oncogene pathway activation [72]. In gene level, oncogenic signaling pathways in tumor cells, such as IFN-γ/JAK2/IFN, ALK/STAT3, PI3K, and MEK/ERK/STAT1 can activate PD-L1 expression [72-74]. Chen et al. demonstrated that the expression of PD-L1 was higher in EGFR-mutant NSCLC cell lines than that in cell lines with wild-type EGFR. Three models of EGFR activation including EGF stimulation, EGFR-19del and EGFR-L858R mutation can increase PD-L1 expression *via* p-ERK1/2/p-c-Jun and p-AKT/p-S6 pathway [72]. Moreover, exposure to EGFR inhibitor-TKIs can decrease the expression of PD-L1 [73]. Ota's research has shown that the level of PD-L1 expression in NSCLC cells positive for EML4-ALK is higher than those in wild-type for both EGFR and ALK. EML4-ALK can upregulate PD-L1 expression at the mRNA and protein levels in Ba/F3 *via* activating PI3K-AKT [74]. The receptor tyrosine kinase c-Met binding to its ligand hepatocyte growth factor can remarkably promote the expression of PD-L1 *via* activating Ras-PI3K signaling pathway, and this condition can be disrupted following treatment of the cells with pharmacological inhibitors of c-Met [75]. Moreover, Parsa et al. identified that the loss of phosphatase and tensin homolog (PTEN) and the resulting activation of phosphatidylinositol-3-OH kinase (PI-3K) pathway enhanced PD-L1 expression in glioma [72, 76].

**Figure 2:**
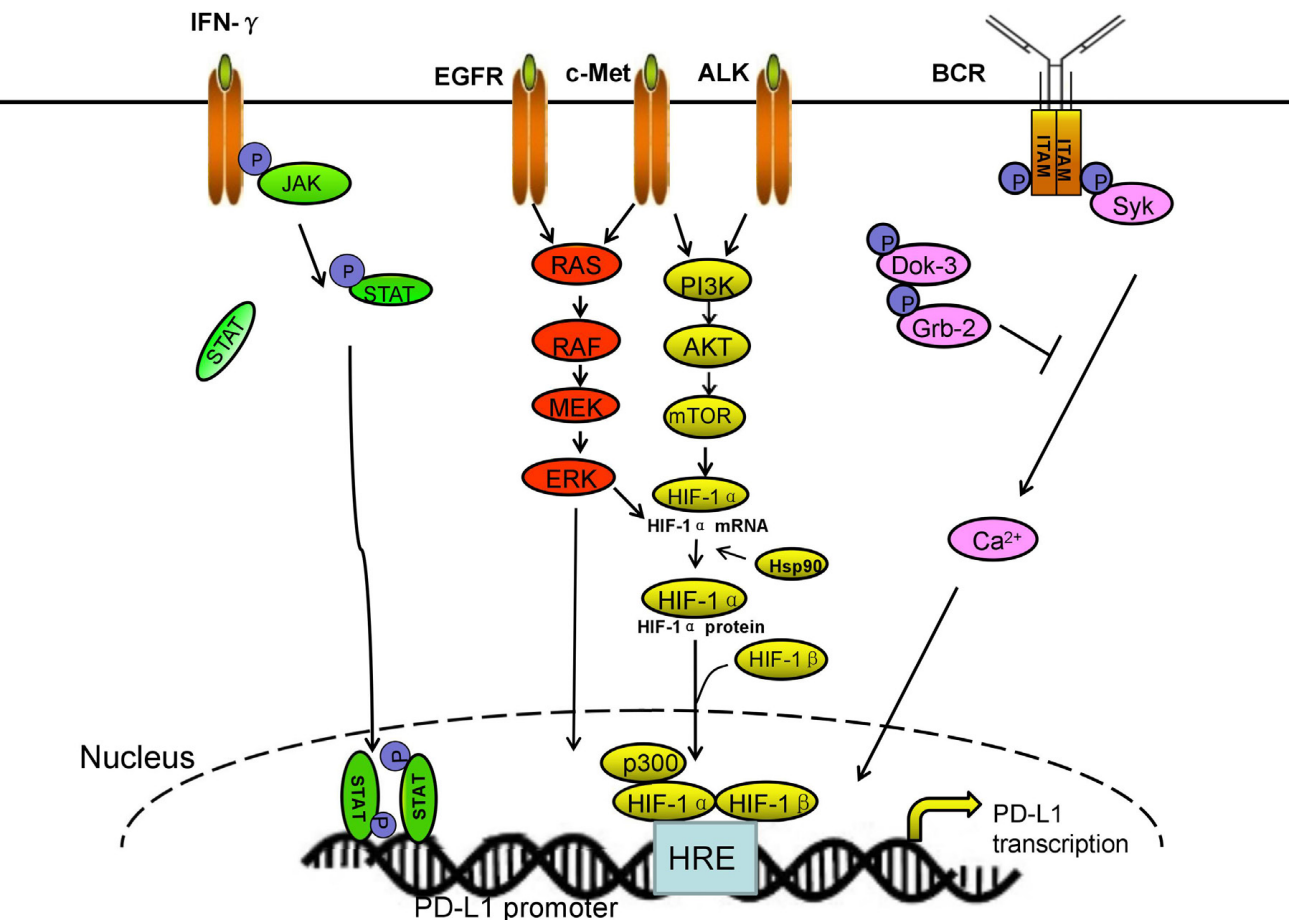
The main signal pathways of PD-L1 transcriptional regulation Multiple pathways promote PD-L1 expression on transcriptional level.

In addition, hypoxia-inducible factor-1α (HIF-1α) is also a major regulator of PD-L1 mRNA and protein expression. Hypoxia causes a rapid, dramatic, and selective upregulation of PD-L1 in splenic myeloid-derived suppressor cells (MDSCs), macrophages, DCs, and tumor cells in tumor-bearing mice through HIF-1α. Binding of HIF-1α to a transcriptionally active hypoxia-response elements (HREs), HRE-4 and HRE-1, at two different HRE sites in the PD-L1 proximal promoter in hypoxic MSC-1 cells is comparable with their binding to an established HRE in VEGF, lactate dehydrogenase A (LDHA), and Glut1 genes [77]. Boes et al. identified that TLR3 triggering results in remarkably upregulation of PD-L1 on neuroblastoma cells. In addition, docking protein 3 (DOK3) increases PD-1 ligand expression through abrogating the intensity of calcium signaling at the transcriptional level. DOK3 recruits growth factor receptor-bound protein 2 (Grb2). Together, DOK3 and Grb2 sequester Bruton tyrosine kinase and diminish PLCγ 2 activation and, thereby, attenuate calcium signaling. Calcineurin inhibition study demonstrated that calcium signaling directly negatively regulates PD-1 ligand gene expression [78-82]. Above all, the main signaling pathways of PD-L1 transcriptional regulation are shown in Figure 2.

Despite recent study efforts on PD-L2, the transcriptional regulation of the ligand is yet to be completely clarified [63]. Several inflammatory cytokines, especially IL-4, upregulate the expression of PD-L2 on DCs and macrophages [34, 83, 84].

## FUNCTIONAL IMPLICATIONS OF ALTERED BIOCHEMICAL SIGNALING INDUCED BY PD-1

### PD-1/PD-L1 axis inhibits T cell response

PD-1/PD-L1 suppresses the effector phase of T-cell response. This condition induces immune tolerance during different phases of T-cell responses, such as regulating the threshold for T-cell activation, inhibiting T-cell proliferation, and promoting apoptosis in activated T cells. The mechanism of PD-1/PD-L1 regulation is correlated with dephosphorylation of signaling molecules belonging to TCR pathway and transmission of inhibitory signal to T cells [31, 85-88]. TCR signaling leads to intracellular Ca2+ flux, which activates multiple signaling pathways required for differentiation and activation of T-cell. PD-1 on T-cell binding to PD-L1 or PD-L2 on APC can activate Lck-mediated phosphorylation of cytoplasmic domain tyrosine and recruitment of SHP-2 to the C-terminal tyrosine of PD-1 cytoplasmic domain [27, 89]. SHP-2 and SHP-1 are two highly related tyrosine phosphatases, which dephosphorylate TCR-proximal signaling molecules, such as protein kinase Cθ, CD3ζ, PI3K and zeta-chain-associated protein kinase (ZAP70), and Syk downstream of B cell antigen receptor (BCR), leading to inhibition of downstream signaling [89, 90]. SHP-2 and SHP-1 are recruited to ITSM of PD-1, leading to inhibition of the PI3K/AKT and mitogen-activated protein kinase signaling pathways downstream of the TCR and blockade of cell-cycle progression in the immune cells [91]. Whereas, PD-1/PD-Ls inhibit Ca2+ flux increasing the number of engaged TCRs required to initiate a Ca2+ flux [92].

PI3K/Akt pathway activation increases Glut1 expression and enhances glucose uptake inducing glycolysis and protein synthesis in T cells [93-95]. After PD-1 recruits the SHP-1 and SHP-2 phosphatase, PI3K/Akt pathway was inhibited. This condition inhibits cell survival proteins, such as B-cell lymphoma-xL (Bcl-xL) which is important for the intrinsic apoptotic pathway; decreases expression of glucose transporters on the plasma membrane and general downregulation of glycolytic enzyme activity, which depresses proliferation of T cells, thereby restraining its survival and decreasing protein synthesis; inhibits CD28-mediated activation and triggers chromatin changes so that the promoter regions of IL-2, TNF-α, and IFN-γ are decreased [28, 96]. Downregulation of IL-2 secretion, driven partially through early growth response protein 2 (EGR2), induces CD8+ T and CD4+ T-cell anergy [97, 98].

Moreover, studies revealed that engagement of PD-1 by PD-L1 inhibited PLC-γ1 and RAS activation and suppressed MEK/Erk MAP pathway [99, 100]. CalDAG is activated downstream of PLC-γ1^56^ promote Ras GRP1 activation. Ras GRP1 promotes exchange of Ras-GDP to Ras-GTP, which suppresses MEK/Erk, suppressing T-cell proliferation. The effect of PD-1 on MEK/Erk and MAP kinases was selective because PD-1 ligation did not inhibit the activation of Jnk and p38 MAP kinases [100, 101]. Engagement of PD-1 and PD-L1 inhibits multiple transcription factors of T cells expression, such as GATA-3 and T-bet, repressing T-cell response [10, 102]. T-bet is a master transcription factor, which can downregulate inhibitory receptors and is essential for differentiation and function of Th1 cells, CD8+ T cells [45, 101, 103]. High level of T-bet maintains exhausted CD8+ T cells and represses inhibitory receptor expression [45]. In addition, the patients with increased level of T-bet^+^ lymphocytes in tumor of nest and stroma exhibit longer survival time than the low level of such cells [94, 104].

PD-1 can also suppress cell cycle progression of T cell by influencing various regulators of the cell cycle. However, the mechanism is yet to be totally understood. PD-1 signaling prevents cell cycle progression in the G1 phase by increasing the cyclin-dependent kinase (Cdk) inhibitors p27 kip1 and p15 INK4B and repressing the Cdk-activating phosphatase Cdc25A expression. PD-1 inhibits Skp2 by inhibiting PIK3/Akt and Ras/MEK/ERK signaling leading to increase of p27kip1 and inhibition of Cdk2. Cdk2 restraint decreases phosphorylation of Rb and fails to phosphorylate the checkpoint inhibitor Smad3, which inhibits cell cycle progression from the G1 to the S phase in the Cdk2-specific site enhancing transcriptional activity of Smad3 and upregulating p15^INK4B^ expression and restraining the Cdk-activating phosphatase Cdc25A [105, 106].

**Table 1:** Results of trail using anti-PD-1 or anti-PD-L1 agents.

Target	Agent	Disease	Sample size	ORR	adverse effects	Ref.
PD-1	Pembrolizumab	advanced melanoma	135	31-51%	grade 1 or 2 toxic effects	[34]
		advanced melanoma	173	26%	-	[126]
		advanced melanoma	655	33%	grade 3 or 4 treatment-related AEs occurred in 14%	[128]
		follicular lymphoma	32	66%	grade 1 or 2 toxic effects	[130]
	Nivolumab	advanced cancers	296	-	grade 3 or 4 toxicities in 14%	[130]
		Melanoma	107	31%	grade 3–4 adverse events in 5%	[20]
PD-L1	MPDL3280A	metastatic melanoma	38	29%	-	[135]
		NSCLC, renal cancer	52(NSCLC)	22% (NSCLC)	-	[135]
			55(renal cancer)	13% (renal cancer)		
	BMS- 936559	advanced solid tumors	207	-	9% had severe grade 3 or 4	[136]
		NSCLC	49	10.2%	grade 1 or 2	[136]
	MEDI4736	solid tumors	26	-	grades 1 to 2(34%)	[138]

### PD-1/PD-L1 inhibits tumor-infiltrating lymphocytes (TIL) and increases immuno-suppressive Treg function

Engagement of PD-1 and PD-L1 restrains the proliferation, survival, and effector function of CD8+ CTL and promotes apoptosis of TILs [10, 107]. High level of PD-1 expression along with other inhibitory receptors inhibits functions of TILs and decreases its quantity in tumor microenvironment [35, 83, 108]. In addition, TILs increase expression of PD-L1, tryptophan-catabolizing enzyme indoleamine-2,3-dioxygenase (IDO), and FoxP3+ Tregs in the melanoma tumor microenvironment. This condition indicates that TILs are involved in immune-intrinsic negative feedback loop. IDO is in charge of the dissimilation of tryptophan, and influences immune reactions in several situations. PD-1/PD-L1 interaction increases the level of IDO, which exhausts T cells of essential tryptophan and suppresses its metabolites, leading to inhibition of T-cell activation and increasing the number of regulatory T cell [109, 110].

PD-1 pathway activation not only downregulates effector T-cell function but also increases immuno-suppressive Treg function [63]. PD-L1 expression on non-hematopoietic and hematopoietic cells accelerates Treg development and improves Treg function in immune organs and autoimmune attacked tissues [3, 111]. PD-1^−/−^ conventional CD4+ T cells demonstrated a remarkably diminished tendency toward differentiation into peripherally induced Treg (pTreg) cells, which showed that PD-1 is critical for the extrathymic differentiation of pTreg cells *in vivo* [112]. Previous studies suggested that the number of circulating Tregs of lung cancer patients was nearly twofold compared with healthy controls and the expression levels of PD-1 on Tregs were higher in lung cancer samples than in controls [113]. These results suggest that the PD-1/PD-L1 pathway plays a role in Treg induction and is associated with impaired adaptive immunity. In the tumor microenvironment, PD-1 expressed on Tregs accelerates CD4+ T cells differentiating into Foxp3+ Tregs under the circumstances of CD3 and TGF-β. Foxp3 is a critical transcription factor of Tregs, which suppress Th1 responses [94]. Foxp3+ Tregs is a highly immunosuppressive subset of CD4+ T cells that is critical in s uppressing proliferation and cytokine production of other T cells, inhibiting tumor-specific immune responses and maintaining peripheral immune tolerance in cancer patients [114]. Meanwhile, Treg cells express constitutive high levels of PD-1, which enhance Treg functional response or proliferation and inhibit T cells responses [115, 116].

## ANTIBODY BLOCKADE OF PD-1/PD-L1 ON THE TREATMENT OF TUMORS

### Efficacy of inhibition of the PD-1 pathway

Blocking PD-1 passway successfully improves T-cell responses *in vitro* and promotes tumor regression *in vivo* in animals [117, 118]. *In vitro*, antibody blockade of PD-1/PD-L1 enhances antitumor immune responses by decreasing the number and/or the suppressive activity of regulatory T cells and by rescuing of the activity of effector T cells in tissues and the tumor microenvironment. In addition, PD-1/PD-L1 antibody blockade decreases the percentages of the highly immunosuppressive MDSC population. Likewise, blockade of PD-1 in B cells may also enhance activity of natural killer cells and increase antigen-specific antibody production *via* PD-1 positive (PD-1+) B cells [63, 119]. Several studies on syngeneic mouse tumor models demonstrated that the blockade of PD-1/PD-L1 enhances antitumor activity. The level of effector CD4+ T and CD8+ T cells, B cells, and myeloid-derived suppressor cells increased in tumor in mice with PD-1 blockade injected with B16 melanoma cells; T cell proliferation and cytokine production were also enhanced, and tumor sites recruited more effector cells [120, 121]. Blockade of PD-1/PD-L1 has significant influences on different CD4+ T-cell subsets. PD-1 blockade enhanced production of IFN, IL-2, TNF-α, IL-6, and IL-17 and the reduced production of the Th2 cytokines IL-5 and IL-13 [122, 123]. This condition reveals that PD-1 blockade may promote antitumor activity through tipping the Th1/Th2 balance and through stimulation of Th17 cells.

PD-1/PD-L1 blockade demonstrates suppression of tumor growth and less metastases. In the mouse model of bladder cancer, the antibody blockade of PD-1 can increase the number of circulating tumor-specific CD107a-expressing CD8+ T cells and activated (CD25+ FoxP3-) CD4+ splenocytes, as well as significantly reduces tumor size [124]. Monoclonal antibodies blockade of PD-1/PD-L1 has revealed great effect outcomes for a subset of patients with cancer, especially in PD-L1 positive tumors, such as melanoma, hepatocellular carcinoma, lung, kidney, and esophageal cancers, as well as hematological malignancies [19, 112]. The expression of PD-L1 in tumor cells is related to the response of PD-1/PD-L1 inhibitors and may be proposed as a potentially valuable predictive marker for the responsiveness of various cancers, including malignant melanoma, NSCLC, and RCC to PD-L1 or PD-1 blocking antibodies [125].

### Clinical trials of mAbs to PD-1

Pembrolizumab is a highly selective, humanized monoclonal IgG4-kappa isotype antibody against PD-1, which binds to the PD-1 receptor on T cells and prevents PD-1 binding to its ligands PD-L1 and PD-L2 [126]. Pembrolizumab is the first PD-1 checkpoint inhibitor for advanced melanoma approved by FDA after the CTLA-4 inhibitor-ipilimumab [127]. Pembrolizumab has remarkable anti-tumor activity and treatment-related toxicity is acceptable. A total of 135 patients with advanced melanoma were in phase 1 study of pembrolizumab. Approximately 38%-52% of patients treated with doses ranging from 2 mg/kg every 3 weeks to 10 mg/kg every 2 weeks showed long-lasting objective responses. In addition, 81% of patients survived for at least 1 year from the beginning of treatment. Grade 1 or 2 adverse events were shown in the majority of patients. 13% of patients have shown grade 3 or 4 adverse events. This result indicated that patients with advanced melanoma treated with pembrolizumab result in a high rate of sustained tumor regression [34]. Robert et al. assessed the clinical effect of pembrolizumab. 173 patients with advanced melanoma received pembrolizumab treatment. The follow-up time was 8 months and ORR was 26%. This result suggested that pembrolizumab is an effective treatment option for patients with ipilimumab-refractory advanced melanoma, wherein few effective treatment options are available [126]. Another trail showed that treatment of advanced melanoma patients with pembrolizumab acquired an overall objective response rate of 33%, 12 month progression-free survival rate of 35%, and median overall survival of 23 months; grade 3 or 4 treatment-related adverse events occurred in 14% of patients with advanced melanoma [128].

Nivolumab is also a MAb of PD-1, which has shown positive therapeutic activity and an acceptable safety profile in treating tumors [19]. In December 2014, the U.S. FDA granted an accelerated approval to nivolumab to treat patients with unresectable or metastatic melanoma and disease progression following ipilimumab and if patients are B-Raf proto-oncogene, serine/threonine kinase (BRAF) V600 mutation positive, which is a BRAF inhibitor [129]. Treatment with nivolumab was first reported in 2012 (sponsored by BMS). Overall response rates and median survival were 28% and 24 months, respectively. The 1 and 2 year survival rates were 62% and 43%, respectively [19, 89]. A multi-dose phase I dose-escalation trial extended the above findings. In this study, 296 patients with the same advanced cancers were given nivolumab at doses from 0.1 mg kg^−1^ to 10 mg kg^−1^ every 2 weeks for up to 2 years. Objective responses were observed in 18.4% patients with NSCLC, 27.6% patients with melanoma, and 27.2% patients with RCC. No tumor responses were observed in patients with castrate-resistant prostate cancer (CRPC) or carcinoma of colon and rectum (CRC). Treatment-related adverse events were fatigue, anorexia, nausea, rash, and diarrhea. Grade 3 or 4 toxicities were reported in 14% of patients and evident at all dose levels without obvious dose dependency [130]. A study of 107 patients with melanoma were treated with nivolumab between 2008 and 2012 revealed an overall survival of 16.8 months. The 1 and 2 year survival rates were 62% and 43%, respectively. Objective responses were observed in 31% of patients. The appearance of irAE was 54%, but grades 3 and 4 adverse events were only seen in five patients (5%) [20].

Pidilizumab is a humanized IgG-1 kappa recombinant mAb. It was developed from a murine version, mCT-011 or BAT, that was generated with the immunization of Balb/c mice with membranes of a human B-cell lymphoma cell line [131]. In a Phase I study, 17 patients with hematologic malignancies were treated with escalating doses of pidilizumab (0.2 to 6.0 mg/kg). Of the 17 treated patients no clear toxicity reaction was observed during therapy. 33% patients acquired clinical benefit and one acquired complete remission. The study showed the antibody to be safe and well tolerated in this patient population [132]. In an International Phase II Trial, 66 patients with diffuse large B-cell lymphoma (DLBCL) were treated. Treatment-related adverse events was mild. At 16 months after the first treatment, progression-free survival (PFS) was 0.72 (90% CI, 0.60 to 0.82). Among the 24 high-risk patients, PFS was 0.70 (90% CI, 0.51 to 0.82). The study suggested an on-target *in vivo* effect of pidilizumab [133].

AMP-224 is the first recombinant B7-DC-Fc fusion protein. In a PhaseI trial, patients with advanced solid tumors received low dose cyclophosphamide (CTX) on Day 0, followed by AMP-224 (IV infusion, 0.3 to 30 mg/kg) on Days 1 and 15 of each 28-day cycle. Infusion reactions were observed at higher doses (86% at the 10 mg/kg dose). No drug-related inflammatory adverse events were identified contrary to PD-1 blocking antibodies [17].

### Clinical trials of mAbs to PD-L1

MPDL3280A is a humanized IgG4 anti-PD-L1-specific mAb, which is most effective in patients where the immunity response is suppressed by PD-L1 [134]. In a phase I MPDL3280A trial, 38 patients with metastatic melanoma exhibited an ORR of 29% with a 24 week Progression-Free-Survival (PFS) of 43%. This agent was expanded in 52 patients with NSCLC and 55 patients with renal cancer. ORR were 22% and 13%, respectively. Breakthrough designations have also been granted for the clinical development of nivolumab for resistant Hodgkin's lymphoma and MPDL2380a in advanced bladder cancer [135].

BMS-936559 is also a PD-L1-specific IgG4 mAb, which inhibits the binding of PD-L1 with PD-1 and CD80. A Phase I trial showed good curative effect of BMS-936559. Up to 207 patients with solid tumor were treated with BMS-936559 for 12 weeks (median duration of therapy). Approximately 9% of patients had toxic effects of grades 3 or 4. Complete or partial response were showed in 17 patients [136]. A phase II trial has shown activity in NSCLC patients. Notably, 5 of 49 NSCLC patients had an objective response, and the response lasted for ≥24 weeks in 3 patients. In this study, the adverse reaction belonged to grades 1 or 2, including rash, hypothyroidism, and hepatitis [137].

MEDI4736 is a fully-human anti-PD-L1 antibody, which has a triple mutation in its Fc domain to avoid antibody-dependent cell-mediated cytotoxicity [127]. In a phase I trial, MEDI4736 was administered every 2 or 3 weeks in a 3 + 3 dose escalation in 26 patients with solid tumors. Grade 1 and 2 adverse events were appeared in 34%. Diarrhea, fatigue, rash, and vomiting are the mainly side effects [138]. Their research indicated that MEDI4736 was a promising agent to inhibit malignant processes.

### Anti-PD-1 *versus* anti-CTLA-4 agents

The anti-CTLA-4 and anti-PD-1 treatments are correlated with clinical benefits. However, cancer patients trested with anti-PD-1 agents acquire better PFS and ORR comparied to anti-CTLA-4 treatment. Subgroup analyses demonstrated significant PFS (RR: 0.92 *vs*. 0.74; *P* < 0.00001) and ORR (RR: 0.95 *vs*. 0.76; *P* = 0.0004) improvement with anti-PD-1 treatment compared with anti-CTLA-4 when each was compared with the control treatments [139]. Similarly, treatment with pembrolizumab was better tolerated and demonstrated superior PFS compared with chemotherapy in ipilimumab-refractory melanoma patients enrolled in phase 2 KEYNOTE-002 trial [128]. Moreover, no clinically meaningful differences were noted between pembrolizumab doses. In the randomized phase 3 KEYNOTE-006 trial, pembrolizumab (10 mg/kg every 2 and 3 weeks) had fewer toxicities and significantly improved PFS, OS, and ORR compared with ipilimumab [128]. The ORR of nivolumab treatment in ipilimumab-refractory patients was lower compared with ipilimumab-naive patients, while the ORRs of the two groups were higher than the control group. Subgroup analyses revealed that the survival benefit was significantly high with anti-PD-1 treatment regardless of previous response to ipilimumab treatment, thereby suggesting that nivolumab or pembrolizumab is a good choice as the first-line treatment [139]. Similarly, the CheckMate 067 trial demonstrated that combined treatment with ipilimumab and nivolumab has better ORR compared with nivolumab monotherapy, especially in PD-L1-positive patients [139, 140]. Collectively, a certain patient population may selectively respond to anti-PD-1 treatment and benefit from the combination treatment with anti-CTLA-4 agents and anti-PD-1 agents.

## CONCLUSIONS

The co-inhibitory factor PD-1 binds to its ligands, PD-L1 or PD-L2, to transmit inhibitory signals in T cells and anti-apoptotic signals in tumor cells. Thus, PD-1 binding is characterized as one of the major mechanisms of tumor immune escape. Furthermore, blockade of the PD-1/PD-L1 interaction may help restore anti-tumor immunity by several ways, such as the increase of TILs that restrain Treg function and increase the cytokine secretion. Some recent clinical trials on the antibody blockade of PD-1 and PD-L1 also demonstrated effective response. Despite several previous studies on the important role of PD-1 in inhibiting T-cell activation, the biochemical signaling effects of PD-1 are yet to be fully understood. Therefore, further studies on PD-1 are needed. When the checkpoint PD-1 is better understood, cancer immunotherapy will be more effective.
